# Adaptive Response to DNA-Damaging Agents in Natural *Saccharomyces cerevisiae* Populations from “Evolution Canyon”, Mt. Carmel, Israel

**DOI:** 10.1371/journal.pone.0005914

**Published:** 2009-06-15

**Authors:** Gabriel A. Lidzbarsky, Tamar Shkolnik, Eviatar Nevo

**Affiliations:** Institute of Evolution, Department of Evolutionary and Environmental Biology, Faculty of Science and Science Education, University of Haifa, Haifa, Israel; University of Missouri-Kansas City, United States of America

## Abstract

**Background:**

Natural populations of most organisms, especially unicellular microorganisms, are constantly exposed to harsh environmental factors which affect their growth. UV radiation is one of the most important physical parameters which influences yeast growth in nature. Here we used 46 natural strains of *Saccharomyces cerevisiae* isolated from several natural populations at the “Evolution Canyon” microsite (Nahal Oren, Mt. Carmel, Israel). The opposing slopes of this canyon share the same geology, soil, and macroclimate, but they differ in microclimatic conditions. The interslope differences in solar radiation (200%–800% more on the “African” slope) caused the development of two distinct biomes. The south-facing slope is sunnier and has xeric, savannoid “African” environment while the north-facing slope is represented by temperate, “European” forested environment. Here we studied the phenotypic response of the *S. cerevisiae* strains to UVA and UVC radiations and to methyl methanesulfonate (MMS) in order to evaluate the interslope effect on the strains' ability to withstand DNA-damaging agents.

**Methodology/Principal Findings:**

We exposed our strains to the different DNA-damaging agents and measured survival by counting colony forming units. The strains from the “African” slope were more resilient to both UVA and MMS than the strains from the “European” slope. In contrast, we found that there was almost no difference between strains (with similar ploidy) from the opposite slopes, in their sensitivity to UVC radiation. These results suggest that the “African” strains are more adapted to higher solar radiation than the “European” strains. We also found that the tetraploids strains were more tolerant to all DNA-damaging agents than their neighboring diploid strains, which suggest that high ploidy level might be a mechanism of adaptation to high solar radiation.

**Conclusions/Significance:**

Our results and the results of parallel studies with several other organisms, suggest that natural selection appears to select, at a microscale, for adaptive complexes that can tolerate the higher UV radiation on the “African” slope.

## Introduction

Natural populations of most organisms, especially unicellular microorganisms, are constantly exposed to harsh environmental factors which have the ability to affect their growth and survival. UV radiation is one of the most important physical parameters which influences yeast growth in nature [1]–[3]. The “terrestrial” UV radiation is composed of UVA (315–400 nm) and UVB (290–320 nm). Though the majority of UVB radiation (∼95%) is blocked in the atmosphere, it has a significant contribution to the biological effects of UV radiation since it is absorbed by cellular DNA. UVB radiation creates two types of photo-dimers between adjacent pyrimidine bases: cyclobutane-pyrimidine dimers (CPDs) and 6–4 photoproducts (6–4pp) [1]–[3]. UVA radiation is poorly absorbed by cellular DNA but can still damage the cell by indirect photosensitizing reactions which lead to the production of free radicals, singlet oxygen, and eventually CPDs [2], [3]. Both types of products disturb the conformation of the DNA helix, causing bends which inhibit or hinder the progress of DNA and RNA polymerases, thereby interfering with DNA replication and transcription. Methyl methanesulfonate (MMS) is an alkylating agent frequently used in mutagenesis and recombination experiments. MMS changes guanine (to 7-methylguanine) and adenine (to 3-methlyladenine) causing base mispairing and replication blocks, respectively [4].

“Evolution Canyon” in Mount Carmel, Israel (32°24′N, 34°58′E) is a natural microsite fitting for evolutionary studies of interslope adaptive divergence and incipient sympatric speciation across life [5]–[8]. The opposing slopes of this canyon are separated by only 100 m at the bottom and 400 m at the top, and share the same geology, soil, and macroclimate, but they differ sharply in microclimatic conditions [9]. The differences in solar radiation between the two slopes of the canyon (200%–800% more on the “African” slope) caused the development of two distinct biomes. The south-facing slope (“African” slope, AS) is sunnier and has tropical, xeric, savnnoid “African” environment while the north-facing slope (“European” slope, ES) is represented by less-exposed, temperate, “European” macquis forest environment [9]. “Evolution Canyon” is explored as an evolutionary model system for the processes of interslope adaptive responses and incipient sympatric speciation in different organisms from bacteria to fungi, plants, and animals. These studies have found interslope divergence in phenotypic and genotypic characteristics between populations isolated from the “European” slope and the “African” slope at “Evolution Canyon” [5]–[8], [10]–[18], [20]–[21] particularly in studies dealing with radiation, temperature, drought, and oxidative stress, which are the most important stresses triggered by solar radiation. Lupu et al. (2004) studied thermotolerance in *Drosophila melanogaster* and showed that AS lines displayed a higher thermotolerance compared with the ES lines. They also found that “African” lines of barley were more efficient in repairing DNA double-strand breaks during high temperature stress than “European” barley lines [16]. Miyazaki and her colleagues (2003) studied the phenotypic and genotypic responses of natural strains of *Saccharomyces cerevisiae* to oxidative stress and found differences in gene-expression patterns between strains from the different slopes [17]. Singaravelan et al. (2008) showed that melanin concentration in *Aspergillus niger* conidia was significantly higher in sunny AS than in shady ES strains and that it is positively correlated with the culturability of the conidia after UVA radiation [20].

In this work we used 46 natural strains of *Saccharomyces cerevisiae* isolated from several populations from the opposite slopes at the “Evolution Canyon” microsite [12], [22] across a gradient of UV radiation, temperature and drought. Our objective was to study their phenotypic response to UVA and UVC radiations and to MMS in order to evaluate the interslope effect on the strains' ability to withstand DNA damaging agents. We expected to see, and indeed found, diverse adaptive phenotypic responses among the natural strains, revealing a correlation between microclimate and the ability to survive the DNA damaging stresses.

## Methods

### Strains and media

**Figure 1:**
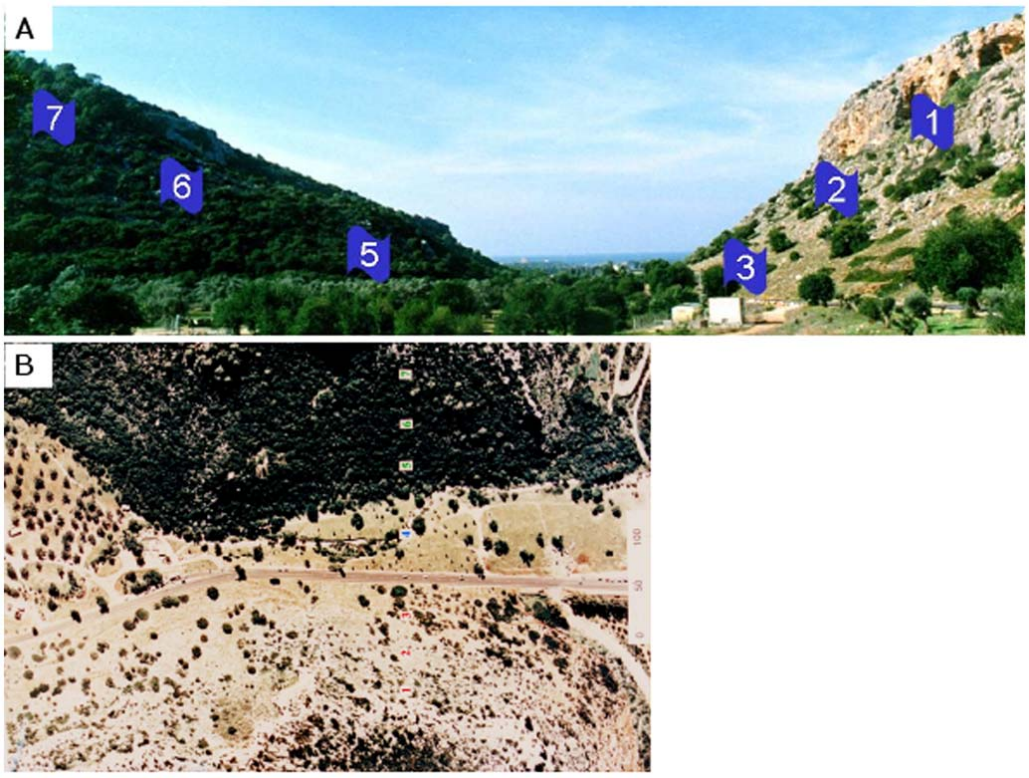
Pictures of “Evolution Canyon”. (A) A cross section of “Evolution Canyon” (lower Nahal Oren, Mt. Carmel). The right slope is the south-facing, “African”, xeric slope, and the left slope is the north-facing, “European”, temperate slope. The numbers represent the populations (sites). (B) An air-view of the canyon. The dark area is the “European” forested slope; the light area is the “African” savannoid slope.

Forty-six *S. cerevisiae* strains (Table S1) were isolated from seven populations (sites) across the two slopes of “Evolution Canyon” (Fig. 1). Isolation and physiological identification was done by Nagornaya S. et al. in 2003 [22]. Further molecular identification and the determination of ploidy levels, was done by Katz-Ezov et al. in 2006 [12]. Collection sites are numbered 1–7; sites 1, 2, and 3 (AS1, AS2, and AS3) are located on the tropical, xeric, and savannoid “African” slope (AS), and sites 5, 6, and 7 (ES5, ES6, and ES7) are located on the temperate, mesic, and forested “European” slope (ES). A commercial lab strain, S288C [23] was used as a control. Strains were grown on liquid and solid YPD (2% glucose, 2% peptone, 1% yeast extract); 2% agar was added to solid media.

### UV survival assays

Pre-cultures were made by taking colonies from fresh over-night cultures to liquid YPD (10 ml in 50 ml tubes). The pre-cultures were incubated (30°C, 175 rpm) until saturation (10^8^ cells/ml). Ten microliters were taken from the pre-culture and were added to 10 ml fresh YPD to create a starter culture. The starter was grown for 10 hours until mid-log phase (∼1×10^7^ cells/ml). Mid-log cultures were serially diluted and spread on solid YPD plates. The serial dilutions were determined in preliminary assays. The dilutions that were used produced 50–250 colonies per plate (10^−5^ for the treated plates, 20^−5^ for the control plates). Irradiation treatment was applied by exposure of the plates to radiation composed of UVA plus 1.4% UVB for 40 minutes with a dose of 60,000 mW*s/cm2 (Philips “Cleo Performance” 40 W tanning Sunlamp) and UVC lamps for 30 seconds exposure (Philips TUV sterilization lamp). After the irradiation, assay and untreated plates were incubated for 48 hours (30°C). The percentage of survival was calculated by dividing the number of colonies in an assay plate by the number of colonies in the control plate.

### MMS sensitivity assays

Samples of 7 Diploid strains and 7 tetraploid strains from each slope were tested for their sensitivity to methyl methanesulfonate (MMS). Mid-log cultures were prepared as for the UV assays. The cultures were serially diluted and 5-microliter drops were spotted on solid YPD plates containing different concentrations of MMS, which were prepared 12 hours before each experiment. The plates were photographed after 48 hours of incubation at 30°C. The experiment was performed three independent times for each strain.

### Statistics

Ten plates representing as many sites as possible were irradiated simultaneously. For each strain, every experiment contained two technical replicates (plates), which represented 2 starter cultures originated from the same colony (same pre-culture). Each starter was treated as mentioned above, and the produced plates were irradiated one after the other. Independent experiments (biological repeats) were done at different days, with fresh colonies and fresh media. 2–3 independent experiments were performed for each strain. For every individual strain, we calculated survival averages for each independent experiment (biological repeat) and these data were used to calculate the P values of the different slopes and the different ploidy clusters. The averages of the independent experiments were also used to calculate the “total” average of each individual strain. P values were analyzed by Kruskal-Wallis and Mann-Whitney tests using SPSS software. The reproducibility of the results, for each individual strain, was assessed by comparing the averages of the strain's independent assays and testing them for statistically significant differences (Kruskal-Wallis test, SPSS software). For all strains, all the averages were not significantly different (P>0.05; see list of P-values in table S2).

## Results

### Exposure to UVA

#### Interslope comparison

**Figure 2:**
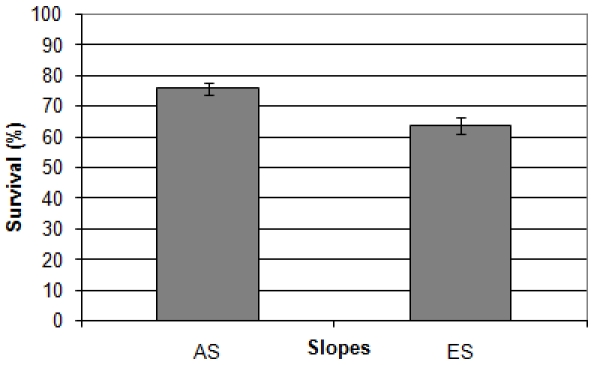
Total averaged survival score of the “African” (AS) and “European” (ES) strains (UVA radiation). n = 23 for each slope. The bars represent standard errors. The averages are significantly different (Mann-Whitney test, p<0.001).

The total averaged survival rate of the “African” slope strains (sites AS1, AS2, and AS3) was significantly (Mann-Whitney test, p<0.001) higher than the total averaged survival rate of the “European” slope strains (sites ES5, ES6, and ES7) (Fig. 2).

#### Ploidy comparison

**Figure 3:**
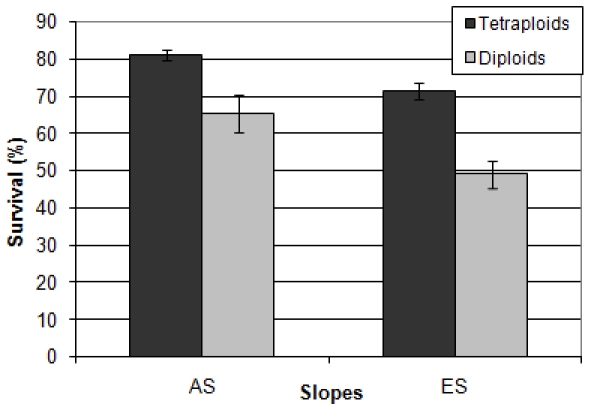
Averaged survival rates of the “African” (AS) and “European” (ES) diploid and tetraploid strains (UVA radiation) . The diploids sample size is 7 for each slope. The tetraploids sample size is 14 for the AS and 13 for the ES. Bars represent standard errors. The survival rate of the “African” diploids was significantly higher than the survival rate of the “European” diploids. (Mann-Whitney test, p = 0.004). The survival rate of the “African” tetraploids was significantly higher than the survival rate of the “European” tetraploids. (Mann-Whitney test, p = 0.001). The survival rate of the “African” diploids was not significantly different than the survival rate of the “European” tetraploids (Mann-Whitney test, p = 0.81).

To see whether the ploidy level had an impact on the strains' survival, we analyzed the results comparing ploidy groups. The results showed that the ploidy level did affect the survival of the strains, as the total survival rates of the tetraploid and triploid were significantly higher than the total survival rates of the diploid strains (Kruskal-Wallis test, p<0.001), although there was no significant difference in the survival rates of the tetraploid and the triploid strains (Mann Whitney test, p = 0.54). When the diploid and the tetraploid strains from both slopes were compared separately, results showed that the survival rates of the “African” diploid and tetraploid strains were significantly higher than the survival rates of the matching “European” strains (Fig. 3 and Figs. S1-S2). On both slopes, the tetraploid strains were significantly more resilient to UVA radiation than the neighboring diploid strains (Mann Whitney test, AS: p = 0.02, ES: p<0.001), however, there was no significant difference between the “African” diploids and the “European” tetraploids (Man-Whitney test, p = 0.81).

### Exposure to UV-C

**Figure 4:**
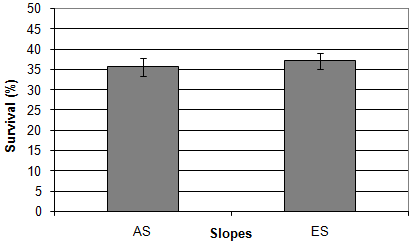
Total averaged survival score of the “African” (AS) and “European” (ES) strains (UVC radiation). n = 23 for each slopes. The bars represent standard errors. The averages are not significantly different (Mann-Whitney test, p = 0.436).

**Figure 5:**
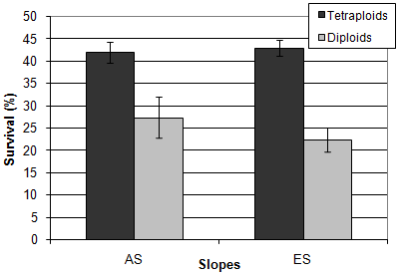
Averaged survival rates of the “African” (AS) and “European” (ES) diploid strains (UVC radiation). The diploids sample size is 7 for each slope. The tetraploids sample size is 14 for the AS and 13 for the ES. Bars represent standard errors. The survival rate of the “African” diploid is not significantly different than the survival rate of the “European” diploids. (Mann-Whitney test, p = 0.442). The survival rate of the “African” tetraploids is not significantly different than the survival rate of the “European” tetraploids. (Mann-Whitney test, p = 0.4).

**Figure 6:**
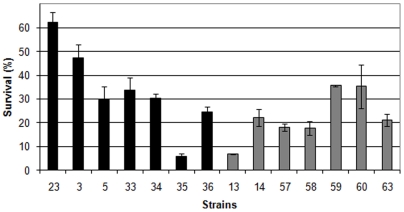
Survival rates of the “African” (AS) and “European” (ES) diploid strains (UVC radiation). The AS strains are colored in black and the ES strains are colored in gray. Bars represent standard errors.

The total averaged survival rate of the “African” slope strains (sites AS1, AS2, and AS3) was nearly the same as the total averaged survival rate of the “European” slope strains (sites ES5, ES6, and ES7) (Fig. 4). As in the UVA assay, the ploidy level had an effect on the survival of the strains. The total survival rates of the tetraploid and triploid was significantly higher than the total survival rates of the diploid strains (Kruskal-Wallis test, p<0.001). The survival rates of the “African” diploid and tetraploid strains were not significantly different than the survival rates of the equivalent “European” strains (Figs. 5–6 and Fig. S3) although the similarity between the survival rates of the diploid strains was contributed by a single irregular strain, which reduced the survival rate of the “African” diploids (Fig. 6). Without this strain, the “African” diploids are significantly more resilient to UVC radiation than the “European” diploids (p = 0.002). As in the UVA assay, for both slopes, the tetraploid strains were significantly more resilient to UVC radiation than the neighboring diploid strains (Mann Whitney test, AS: p = 0.022, ES: p<0.001).

### MMS sensitivity assays

**Figure 7:**
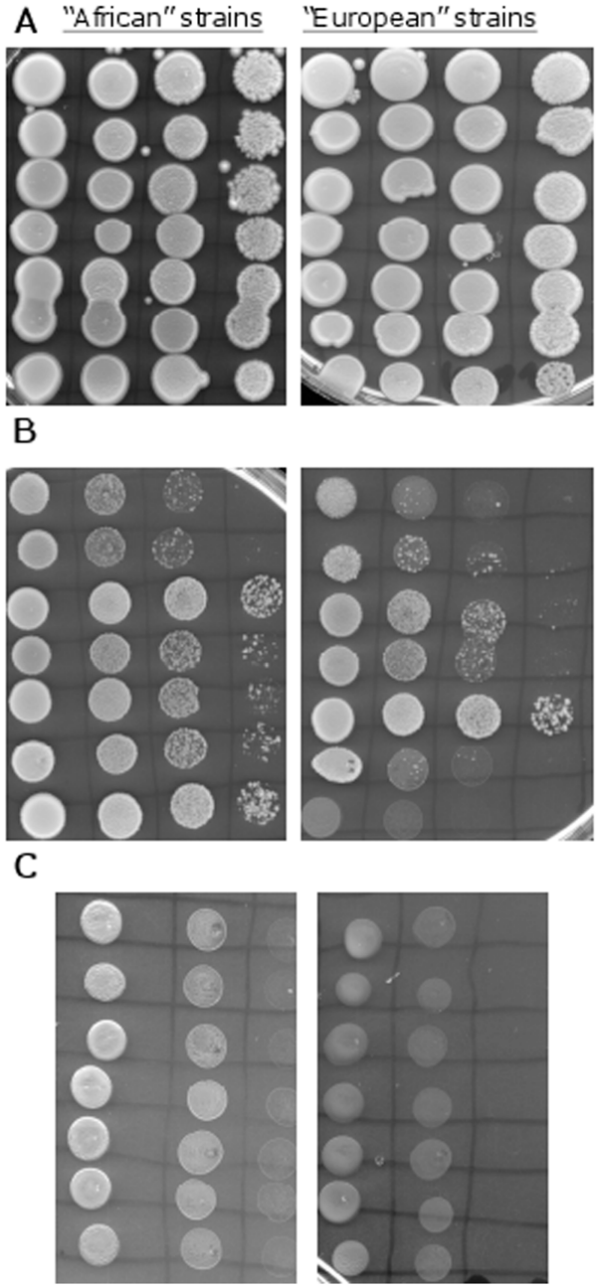
MMS sensitivity assay with diploid strains. In the three pictures the “African” diploid strains are on the left side of the picture, and the “European” diploid strains are on the right (n = 7 for each slope). (A) YPD plates containing 0.01% MMS. (B) YPD plates containing 0.03% MMS. (C) YPD plates containing 0.05% MMS.

**Figure 8:**
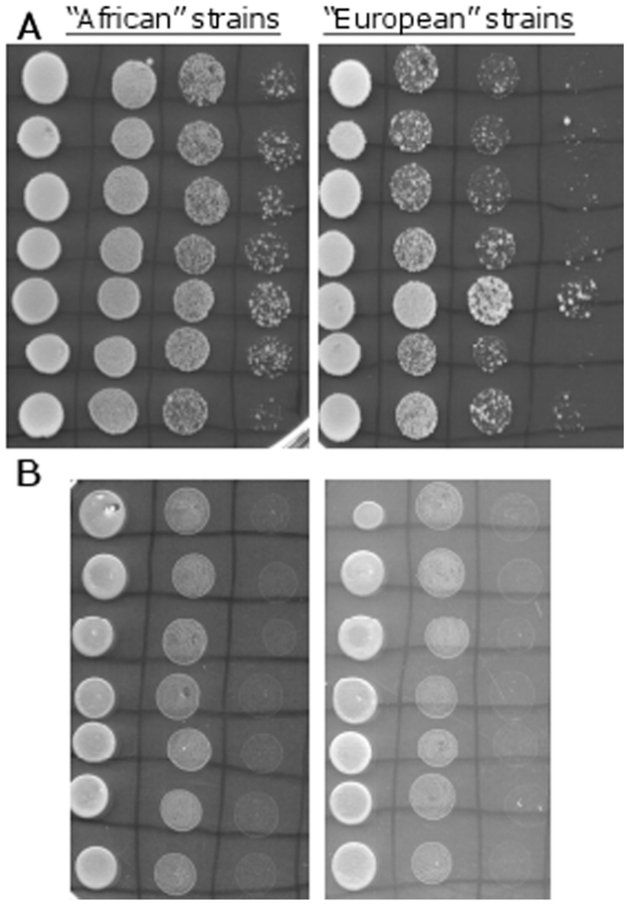
MMS sensitivity assay with tetraploid strains. In the two pictures the “African” tetraploid strains are on the left side of the picture, and the “European” tetraploid strains are on the right (n = 7 for each slope). (A) YPD plates containing 0.03% MMS. (B) YPD plates containing 0.05% MMS.

The “African” and “European” diploid strains showed different sensitivity to different MMS concentrations. All diploid strains were able to grow well on plates containing 0.01% MMS (Fig. 7-A). However, when the diploid strains were challenged by MMS concentration of 0.03% the “African” strains were more resilient than the “European” strains (Fig. 7-B). Similar trend was observed when the tetraploid strains were challenged with 0.03% MMS. The “African” strains grew better than the “European” strains (Fig. 8-A). As in the UV assays, for both slopes, the tetraploid strains were more resilient to 0.03% MMS than the neighboring diploid strains, and the “African” diploid strains were more resilient to 0.03% MMS than the “European” tetraploid strains. All strains, diploid and tetraploid, showed poor growth ability on plates containing 0.05% MMS although the spots created by the “African” strains seemed bigger and rounder (Fig. 7-C and Fig. 8-B)

## Discussion

In the presented work, we tested natural *Saccharomyces cerevisiae* strains for their ability to withstand different DNA damaging agents, UVA and UVC radiations and methyl methanesulfonate (MMS). The studied strains were isolated from “Evolution Canyon” (Nahal Oren, Mt. Carmel, Israel), a well established “natural laboratory” for analyzing interslope adaptive divergence and incipient sympatric speciation across life [8]. The two slopes of the canyon represent two very contrasting biomes created by interslope solar radiation: a sunnier, xeric tropical savannoid “African” slope and a less-exposed, forested shadier temperate “European” slope [5]–[8]. These differences, exhibited in a very small sampled area of 7000 m^2^, are very useful for studying biodiversity, evolution and adaptive processes, especially adaptation to high solar radiation, oxidative stress and drought, as well as incipient sympatric speciation.

### Summary of results

We have found that the strains from the tropical “African” sun-exposed slope were more resilient to radiation composed of UVA and 1.4% UVB than the strains from the temperate “European” slope. The “African” strains were also more tolerant to MMS (especially in the concentration of 0.03%) than the “European” strains. In contrast, we found that there was no difference between strains (with similar ploidy) from the opposite slope, in their sensitivity to UVC radiation, although differences appeared (in diploid strains) when we removed one irregular strain from AS.

### Ploidy level and resistance to UV radiation

We found that the strains from the “African” sun-exposed slope were more resilient to radiation composed of UVA and 1.4% UVB than the strains from the “European” temperate slope. These results suggest an adaptation to tolerate high solar radiation on the exposed AS, as the natural solar radiation contains also a mixture of UVA and 5% UVB [3]. Tetraploid strains were more resilient to this radiation than their neighboring diploid strains. Theoretically, these results were expected from the nature of the damage caused by the radiation – direct and indirect damage to the structure and function of the DNA [1]–[3]. Multiple copies of the DNA allow the cell to recover more easily from the damage. However, former studies have found that ploidy level does not necessarily protect against DNA-damaging agents. Mable and Otto (2001) found that there was no difference in sensitivity between diploids and tetraploids strains of *S. cerevisiae* to the alkylating agent EMS (Ethane Methyl Sulphonate), that all ploidy levels had the same recovery rate, and that the genome of the diploid strains was more stable in the presence of EMS than the genome of the tetraploids strains. They showed a decrease in ploidy level in tetraploids cells and an increase in the ploidy level of the haploid cells (both towards diploidy), while the diploid cells did not change [24]. In contrast to these findings, Sasaki (1992) showed that UV radiation changed ploidy level, but it increased the ploidy level of diploids to tetraploidy [25]. Diploidy is considered more stable than tetraploidy, and most natural *S. cerevisiae* strains are diploids [26], [27], but tetraploidy might have an advantage in coping with radiation stress as the cell is bigger and more robust [25]. In spite of the ploidy effect, the AS diploid strains were as resilient as the tetraploid ES strains. This observation emphasizes the improved ability of the “African” strains to survive high solar radiation by other means than by the ploidy level.

UVA and UVB damage the DNA in different ways. UVA causes indirect oxidative stress via photosensitizing reactions, while UVB causes direct structural damage in the form of cyclobutane–pyrimidine dimers (CPD) and 6–4 photoproducts [3]. The resilience of the “African” strains could suggest an improved capability to repair both kinds of damages. This assumption corroborate the findings that the “African” strains from “Evolution Canyon” are also more resilient to oxidative stress than the “European” strains [17].

The results of the UVC-exposure assays were somewhat different. There were no differences between the strains (with similar ploidy) from the opposite slopes, although, in the case of the diploid strains this was attributed to one irregular strain, and the removal of this strain produced different results. UVC is not a “natural”, “terrestrial” radiation since it is fully absorbed in the atmosphere [3], and this might be the cause for the different results, since terrestrial organisms are not exposed to this radiation and thus cannot adapt to it.

### Ploidy level and interslope MMS resistance

The “African” strains, diploid and tetraploid, were more tolerant to MMS (in the concentration of 0.03%) than the “European” strains. DNA damage caused by MMS and oxidative stress is repaired mostly by the base excision repair (BER) pathway and DNA alkyltransferases, while UVB induced damage is repaired mostly by nucleotide excision repair pathway (NER) and photolyases [28], [29]. Genes that are involved in DNA repair belong to three epistatic groups labeled RAD3, RAD6 and RAD52. The NER pathway is associated primarily with the RAD3 group, whereas the BER pathway is controlled mainly by RAD6 and RAD52 groups [30], [31]. The results of the sensitivity assay to MMS might indicate that the adaptation of the “African” strains to high solar radiation is attributable to genetic changes in several DNA repair pathways.

### Parallel DNA repair studies at “Evolution Canyon”

Several studies regarding DNA damage response have been done using organisms from the “Evolution Canyon” model, which has been extended to four similar natural labs at the Upper Galilee (EC II), Golan Heights (EC IV) and the Negev desert (EC III), all proved ideal models for studies of interslope adaptation and incipient sympatric speciation [8]. Lupu and her colleagues (2004) found that “African” lines of the fruit fly *Drosophila melanogaster* were more tolerant to heat and to DNA damaging agents than the “European” lines. They also found similar results in barley (2006), when “African” lines were more efficient in repairing DNA double-strand breaks during high temperature stress than “European” lines [15], [16]. Sikorski and Nevo (2005) found in the soil bacterium *Bacillus simplex* that the levels of UVC sensitivity and spontaneous mutation rate were correlated with ecological niches and phylogeny thereby pointing not only to adaptation but also to speciation [19]. Singaravelan et al. (2008) showed that melanin concentration in the soil microfungus *Aspergillus niger* conidia and tolerance to UVA was significantly higher in the “African” yeast strains than in the “European” strains [20]. These results support our hypothesis that the “African” strains have adapted to their harsher microclimatic environment as compared to the “European” environment, and therefore are more suitable to survive high amounts of solar radiation than the matching “European” strains. Natural selection appears to select, at a microscale, for adaptive complexes in diverse taxa across life (including yeast) that can tolerate the higher UV radiation, temperature and drought on the “African” slope.

## Supporting Information

Figure S1 Survival rates of the African (AS) and European (ES) diploid strains (UVA radiation). The AS strains are colored in black and the ES strains are colored in gray. Bars represent standard errors.
(0.02 MB TIF)

Figure S2 Survival rates of the African (AS) and European (ES) tetraploi strains (UVA radiation). The AS strains are colored in black and the ES strains are colored in gray. Bars represent standard errors.
(0.05 MB TIF)

Figure S3 Survival rates of the African (AS) and European (ES) tetraploid strains (UVC radiation). The AS strains are colored in black and the ES strains are colored in gray. Bars represent standard errors.
(0.04 MB TIF)

Table S1 list of the strians used in the study
(0.06 MB DOC)

Table S2 P-values of the reproducibility assessment tests
(0.01 MB DOC)
