# Supplementary figures and images for: Adaptive Response to DNA-Damaging Agents in Natural Saccharomyces cerevisiae Populations from “Evolution Canyon”, Mt. Carmel, Israel
Source: PLoS One. 2009 Jun 15;4(6):e5914. doi: 10.1371/journal.pone.0005914 (PMC2690839; doi:10.1371/journal.pone.0005914)

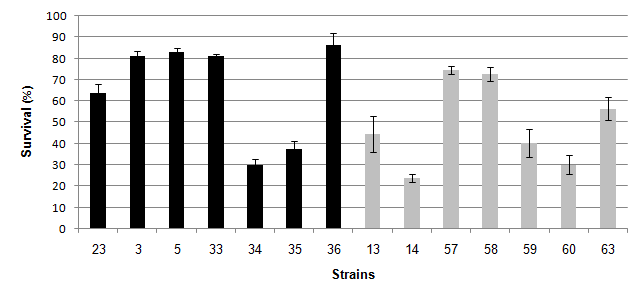

Supplement: Figure S1 — Survival rates of the African (AS) and European (ES) diploid strains (UVA radiation). The AS strains are colored in black and the ES strains are colored in gray. Bars represent standard errors. (0.02 MB TIF) [file pone.0005914.s001.tif]

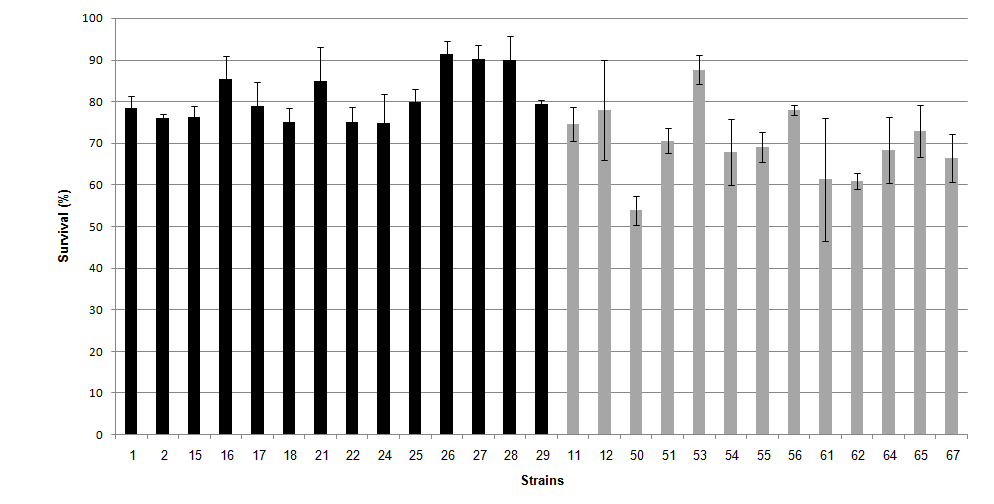

Supplement: Figure S2 — Survival rates of the African (AS) and European (ES) tetraploi strains (UVA radiation). The AS strains are colored in black and the ES strains are colored in gray. Bars represent standard errors. (0.05 MB TIF) [file pone.0005914.s002.tif]

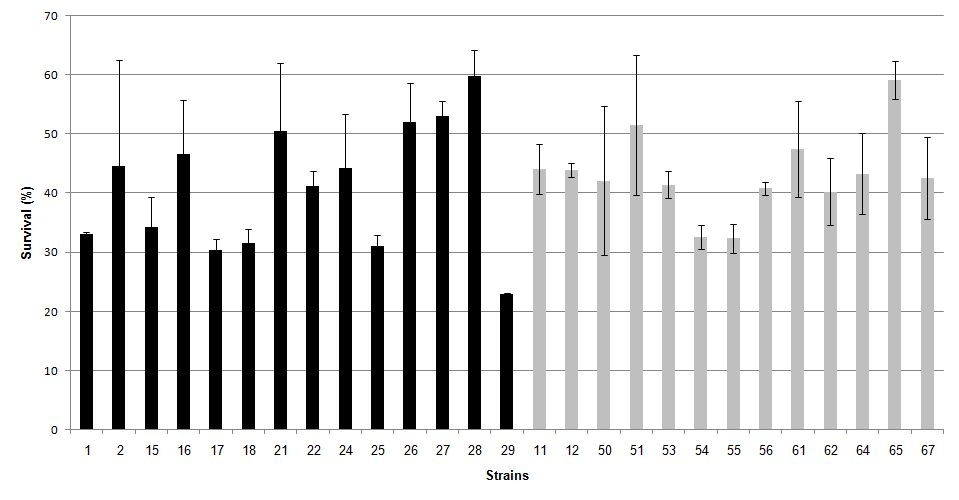

Supplement: Figure S3 — Survival rates of the African (AS) and European (ES) tetraploid strains (UVC radiation). The AS strains are colored in black and the ES strains are colored in gray. Bars represent standard errors. (0.04 MB TIF) [file pone.0005914.s003.tif]
