# Supplementary material for: Adaptive Response to DNA-Damaging Agents in Natural Saccharomyces cerevisiae Populations from “Evolution Canyon”, Mt. Carmel, Israel
Source: PLoS One. 2009 Jun 15;4(6):e5914. doi: 10.1371/journal.pone.0005914 (PMC2690839; doi:10.1371/journal.pone.0005914)
Supplement: Table S1 — list of the strians used in the study (0.06 MB DOC) [file pone.0005914.s004.doc]

**Table S1. List of strains used in the study:**

| **Strain** | **Populations (sites)** | **Ploidy (n)** |
| --- | --- | --- |
| S288C | Lab | 1 |
| St1 | "African" 1 | 4 |
| St2 | "African" 1 | 4 |
| St15 | "African" 1 | 4 |
| St16 | "African" 1 | 4 |
| St17 | "African" 1 | 4 |
| St18 | "African" 1 | 4 |
| St19 | "African" 1 | 3 |
| St20 | "African" 1 | 3 |
| St21 | "African" 2 | 4 |
| St22 | "African" 2 | 4 |
| St23 | "African" 2 | 2 |
| St24 | "African" 2 | 4 |
| St25 | "African" 2 | 4 |
| St26 | "African" 2 | 4 |
| St27 | "African" 2 | 4 |
| St28 | "African" 2 | 4 |
| St29 | "African" 2 | 4 |
| St3 | "African" 3 | 2 |
| St5 | "African" 3 | 2 |
| St33 | "African" 3 | 2 |
| St34 | "African" 3 | 2 |
| St35 | "African" 3 | 2 |
| St36 | "African" 3 | 2 |
| St11 | "European" 5 | 4 |
| St12 | "European" 5 | 4 |
| St50 | "European" 5 | 4 |
| St51 | "European" 5 | 4 |
| St52 | "European" 5 | 3 |
| St53 | "European" 5 | 4 |
| St54 | "European" 5 | 4 |
| St13 | "European" 6 | 2 |
| St14p | "European" 6 | 2 |
| St55 | "European" 6 | 4 |
| St56 | "European" 6 | 2 |
| St57 | "European" 6 | 2 |
| St58 | "European" 6 | 2 |
| St59 | "European" 6 | 2 |
| St60 | "European" 6 | 2 |
| St61 | "European" 7 | 4 |
| St62 | "European" 7 | 4 |
| St63 | "European" 7 | 2 |
| St64 | "European" 7 | 4 |
| St65 | "European" 7 | 4 |
| St66 | "European" 7 | 3 |
| St67 | "European" 7 | 4 |
| St68 | "European" 7 | 3 |
