# Supplementary material for: Adaptive Response to DNA-Damaging Agents in Natural Saccharomyces cerevisiae Populations from “Evolution Canyon”, Mt. Carmel, Israel
Source: PLoS One. 2009 Jun 15;4(6):e5914. doi: 10.1371/journal.pone.0005914 (PMC2690839; doi:10.1371/journal.pone.0005914)
Supplement: Table S2 — P-values of the reproducibility assessment tests (0.01 MB DOC) [file pone.0005914.s005.doc]

**Table S2**. **P-values** (reproducibility assessment, Kruskal-Wallis test):

| **Strain** | **UVA experiment** | **UVC experiment** |
| --- | --- | --- |
| St3 | 0.86 | 0.564 |
| St5 | 0.368 | 0.06 |
| St23 | 0.362 | 0.655 |
| St33 | 0.317 | 0.248 |
| St34 | 0.368 | 0.374 |
| St35 | 0.223 | 0.222 |
| St36 | 0.497 | 0.275 |
| St13 | 0.165 | 0.9 |
| St14 | 0.365 | 0.121 |
| St57 | 0.85 | 0.564 |
| St58 | 0.221 | 0.06 |
| St59 | 0.284 | 0.85 |
| St60 | 0.532 | 0.121 |
| St63 | 0.362 | 0.407 |
| St1 | 0.368 | 0.827 |
| St2 | 0.862 | 0.083 |
| St15 | 0.532 | 0.344 |
| St16 | 0.7 | 0.202 |
| St17 | 0.407 | 0.564 |
| St18 | 0.368 | 0.554 |
| St21 | 0.165 | 0.121 |
| St22 | 0.368 | 0.564 |
| St24 | 0.333 | 0.083 |
| St25 | 0.8 | 0.248 |
| St26 | 0.8 | 0.18 |
| St27 | 0.261 | 0.407 |
| St28 | 0.333 | 0.439 |
| St29 | 0.85 | 0.827 |
| St11 | 0.407 | 0.2 |
| St12 | 0.259 | 0.825 |
| St50 | 0.368 | 0.121 |
| St51 | 0.317 | 0.139 |
| St53 | 0.285 | 0.155 |
| St54 | 0.165 | 0.766 |
| St55 | 0.439 | 0.513 |
| St56 | 0.9 | 0.564 |
| St61 | 0.121 | 0.083 |
| St62 | 0.317 | 0.655 |
| St64 | 0.407 | 0.7 |
| St65 | 0.407 | 0.7 |
| St67 | 0.317 | 0.513 |
